# Correlating Access to Primary Medical Care and Veterinary Care Providers: A Novel Application of Spatial Gravity Modelling

**DOI:** 10.3390/vetsci10090565

**Published:** 2023-09-11

**Authors:** Sue M. Neal

**Affiliations:** 1 Department of Political Science, Arkansas State University, Jonesboro, AR 72467, USA; sneal@astate.edu; 2 Veterinary Care Accessibility Project, Rochester, MI 48306, USA; 3 Center for GIS and Spatial Analysis, West Chester University, West Chester, PA 19383, USA

**Keywords:** One Health, access to care, public health access, veterinary care access, floating catchment, E2SFCA

## Abstract

**Simple Summary:**

Access to care is a challenge for both human medicine and animal medicine. The One Health paradigm recognizes that human, animal, and environmental health are interconnected. Focusing on the human and animal health aspects of this, this research evaluated whether there was a correlation between lack of access to primary care physicians and veterinary medical staff in a four-county study area in Alabama, USA. The research advanced the novel application of a spatial gravity model to take into account the impact of distance from provider on supply and demand for services. The research found that areas that had low access to primary care physicians also had low access to veterinary care. The implications for this work include suggestions for increased collaboration to resolve access challenges for both humans and animals and also details possible advancements of this type of modelling applied in a One Health perspective.

**Abstract:**

Increasingly, health professionals are recognizing the need for a more holistic, or One Health, approach to wellness. Companion animals share the lives and homes of many Americans, and yet little research exists on the intersection of disparities of access to veterinary care and access to human health care. This research aims to fill that gap by exploring the use of a spatial model that identifies the relationship of supply and demand while also considering reductions as a function of travel time to a care facility. Four counties in Alabama were analyzed at the census tract level to determine the supply of primary care physicians and veterinary care providers. This research provides a unique application of the Enhanced Two-Step Floating Catchment Area model by independently examining access to each type of care and then comparing both access supply values at the census level. Results indicated a statistically significant positive relationship between access to both types of care, implying areas with lower access to primary care have concomitantly low access to veterinary care. Implications for practice include the introduction of this methodological approach, identification of future parameter specification research to improve the approach, and identification of an area of significant concern in the One Health framework. Further, the results can inform solution strategies such as offering integrated care interventions for both humans and their companion animal household members with direct use for policymakers aimed at increasing equitable access to health care across the One Health spectrum.

## 1. Introduction

The COVID-19 pandemic, which may have originated when a virus jumped from a non-human animal to the human population, is a stark reminder that the health of all living things is interconnected. One Health is “a collaborative, multisectoral, and transdisciplinary approach—working at the local, regional, national and global levels—with the goal of achieving optimal health outcomes recognizing the interconnection between people, animals, plants, and their shared environment” [1]. Veterinarians and primary care physicians both serve populations within the One Health domain. The primary care physician (PCP) acts as the entry point for a patient’s health and medical needs, and they can help navigate the healthcare system on the patient’s behalf [2]. Veterinarians are doctors who have been educated to protect the health of animals and people, and also protect the environment, conduct research, and are involved in public health and food safety [3]. Both professions are needed to provide care to the population they serve, but not everyone may have equal access to this care. For example, access to veterinary care has also been identified as the leading issue in animal welfare [4]. The intersection of disparities in access to both veterinary care and primary care manifests in the same regions—further adding to the potential for poor outcomes in these populations. Socioeconomic, health access, and other demographic factors have been shown to operate together in complex systems impacting animals along with people [5,6]. Using a One Health lens, this research focuses on the study population’s access to care for both veterinarians and primary care physicians in four Alabama counties: Montgomery, Autauga, Elmore, and Lowndes, and uses a novel application of the Enhanced Two-Step Floating Catchment Area (E2FSCA) to evaluate the correlation of spatial disparities in access to care for both humans and the companion animals that share their homes. While the environment (including interaction with wild animals) is a key part of One Health, this research makes an important step forward in how to quantify One Health from a care access perspective.

### 1.1. Zoonoses

Pets are a large part of many people’s lives, but studies have shown that physicians do not routinely inquire about patients’ contact with pets or talk about zoonotic diseases, even though patients could have a higher risk of acquiring a pathogen from their pet if they have a compromised or developing immune system [7]. Individuals with a higher risk include children, the elderly, pregnant women, and the immunocompromised [7,8,9]. Zoonotic infections acquired from pets can occur from direct contact with an animal’s mucous membranes, skin, saliva, body fluids or secretions, urine, inhaling aerosols or droplets, ingesting fecal matter, or from scratches or bites [7,8,9]. Although epidemiologic studies and patient surveys have suggested that pet-associated disease is low overall, “companion animals are a potential source for more than 70 human diseases”, and contact with pets is a risk factor for diseases such as bacterial, parasitic, fungal, and viral pathogens [7]. Some examples include *Salmonella*, *Campylobacter*, *Bartonella*, *Brucella canis*, *Capnocytophaga canimorsus*, *Chlamydophila psittaci*, dermatophytes, hookworms, roundworms, *Toxoplasma gondii*, lymphocytic choriomeningitis virus, rabies, cowpox, noroviruses, and multidrug resistant bacteria such as MRSA or *Clostridium difficile*, among others [7,8,9]. The sources of these pathogens include pets like cats, rabbits, dogs, birds, rodents, amphibians, or reptiles [7]. Some pet foods and treats can also be sources of zoonotic pathogens [10]. Veterinarians and healthcare providers don’t usually discuss the risks of zoonotic diseases with each other or patients, creating a professional gap [7]. However, it is estimated that zoonotic diseases account for around 75% of emerging infectious diseases [10]. While animals and humans continue to share environments, zoonotic diseases and their impacts will continue, making the field and mindset of One Health a top priority.

Physical proximity to care providers is an essential part of health care [11,12] and veterinary care [13,14]. Researchers found that improved access to primary care physicians was found to be associated with lower mortality from cancers, heart disease, and all-causes in Washington State [15]. The authors were able to find areas that had primary care physician shortages at finer spatial resolutions which could also help in the implementation of rural residency programs and growing the amount of physicians in the state [15]. Pets and people often share the same environment and similar vector exposures; the presence of concurrent sick pets may prove useful in diagnosing certain illness, as was previously highlighted by a case of Rocky Mountain spotted fever in a patient and her two dogs [7]. This clearly evidences the link between adequate identification of companion animals as a factor in human health. If regions lack access to both veterinary care and primary care, there may be an increased risk for potentially contagious or serious illness to go unrecognized. Additionally, the human–animal bond has been well documented in the literature [16,17,18] and not being able to access care for a companion animal may result in significant distress to the pet owner and family [19]. This research aims to explore the intersection of spatial disparities of lack of physical access to care for both primary care physicians and veterinary professionals in a region of Alabama, United States by applying a type of gravity model.

### 1.2. Floating Catchment Area Model

Modeling spatial access to care has undergone significant evolution over time. Some methods have included kernel density [20], the gravity model [21], and regional availability models [22]. The Floating Catchment model was first put forth by Radke and Mu [23], improved by Lou and Wang [24], and further enhanced by Luo and Qi [25]. The Two-Step Floating Catchment Area (2SFCA) is a spatial model and special case of the classical gravity model [25,26]. It has been used successfully in a number of applications in the field of human health by factoring in distance as an impediment to access to care [27]. In this method, higher results indicate a higher level of access [27]. The model was first advanced by Radke and Mu [23] and later improved several times [23,24,28]. Advantages over a traditional gravity model include ease of interpretation as well as a unique form of service provider-to-population ratio [25]. The basic 2SFCA model follows a two-step process. In the first step, the demand is estimated for each service location and the provider-to-population ratio is subsequently calculated. The second step sums up the ratios of the nearby sites for each population.

The method has been evaluated against other spatial models. For example, one study compared a two-step floating catchment area method and a kernel density method to measure accessibility of dialysis service centers [29]. They found that there was a difference between accessibility ratios between the two methods, with the two-step floating catchment area providing the better ratio and being the recommended method for future use [30]. Further they evaluated the results against observed accessibility and found strong mutual correlation [30].

Further enhancement to the 2SFCA was made by Luo and Qi [25] by applying a distance decay function resulting in the E2SFCA. The E2SFCA functions similarly to the 2SFCA with the addition that in each of the two steps a maximum travel time catchment area is generated for each point. The catchment area is subsequently divided into subzones of travel intervals (these are often 10, 20, and 30 min [31] but can vary with study design, for example in rural areas where travel times may be longer [15,32]. This more accurately represents the reality where lower service usage occurs the further away a care provider is from the patient/client. The authors explain that access to primary care is imperative for population health as it is important for preventive care, and both health service policy makers and planners need to be able to measure accessibility so that they can identify areas with physician shortages and areas that need resources so that resources can be allocated [25]. In the study, to account for distance decay, the E2SFCA method used the Gaussian function weight and two weights for different travel time zones in a catchment. If used for calculating cancer care facility accessibility, the weight could change slowly with distance, while calculating pharmacy service accessibility could cause the weight to decay sharper with distance [25]. Overall, the researchers show that this method can be used to help identify areas of physician shortages to help with determining how to disseminate healthcare resources [25]. While this approach assumes equal access within the subzones, it remains an important improvement.

Floating catchment models have only rarely been used to evaluate access to veterinary care [31]. One recent paper used the E2SFCA to suggest a method to optimize rabies vaccination site locations in Southeastern Brazil [31] and another used the 2SFCA to evaluate spatial access to large animal veterinarians in a region of France [33]. In a review of the literature, the only other paper found to use any form of this method in veterinary care was a paper published in German relating to access to care for companion animals in Switzerland using a FCA model [34]. Broader use of this method can be found in the human health access field. The 2SFCA method has been employed in several studies to estimate spatial access to human healthcare services, for example, [15,20,29,32,35,36,37].

While the E2SFCA methodological approach has been used to evaluate access to health care in previous studies, it has never been applied to examine the intersection of care disparities across multiple species health care in a One Health approach. This research applies the E2SFCA in a novel way. It follows in the methodological footprints of Lou and Qi [25] but extends it in a unique application by analyzing the results for both human access to care and access to veterinary care through measures of correlation in order to determine the relationship between spatial disparities in access to human health simultaneous with access to veterinary care. It draws on a methodology from the field of public health but broadens the use of the approach by examining the question of spatial disparity in supply of care through a One Health lens. This study advances a novel use of the E2SFCA to evaluate the correlation in supply of human primary care providers and veterinary care providers in and around Montgomery Alabama.

## 2. Materials and Methods

### 2.1. Study Area

It is recognized that there are place-based disparities to access to primary care in the state of Alabama [38]. “Access to care is identified as Alabama’s number one health issue in the 2015 State of Alabama Community Health Improvement Plan, with the most significant and universal barrier being access to a Primary Care Physician (PCP)” [39]. Alabama has also been noted as having lower access to veterinary care in a study done to evaluate relative access to veterinary care across the US [14].

**Figure 1:**
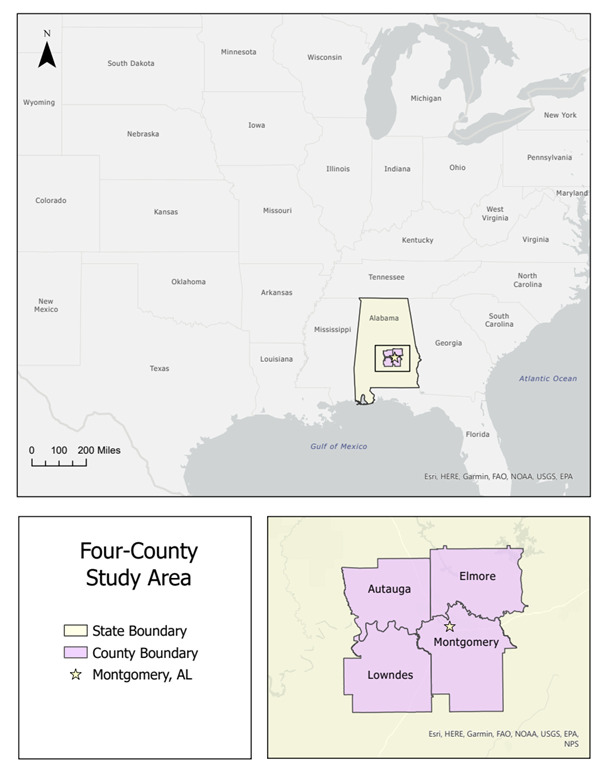
Location of the four-county study area in Alabama, USA.

A four-county area surrounding the Montgomery metropolitan area comprised of Montgomery, Autauga, Elmore, and Lowndes counties was selected as the study area. This region was of interest due to the acknowledged challenges of access to care as discussed. Another benefit of this study area is that it represents a mix of urban and rural settings. Montgomery, a diverse city is situated within the study area and surrounded by more rural outlying counties. Lastly, this area is proximal to Tuskegee University, an HBCU with both health science and veterinary science programs. These features make this area an intriguing potential region for innovative One Health services. Figure 1 shows the location area of the study both nationally and regionally.

### 2.2. Veterinary Service Supply

Veterinary service supply here was conceptualized as the ratio of the number of veterinary clinic employees to the count of households within each census tract. The animal population would provide a metric more similar to that of the human health care equivalent but this data was not available with any degree of reliability at this small of a scale [40]. Veterinary clinics were defined using the North American Industry Classification System. The North American Industry Classification System (hereafter NAICS) provides a standardized method for classifying industries across the continent of North America. The NAICS code 541940 was used as it identifies facilities where licensed veterinary practitioners engage in medical, dental, or surgical care. While the broad code also includes facilities engaging in diagnostic testing, those were removed from this dataset because they do not directly engage in service to the public. All businesses falling under this code were identified for the study area using the Earth Sciences Research Institutes ArcGIS Online tool. In addition to location, the database included the number of individuals employed at each location. The total number of employees were used, rather than the number of veterinarians because the additional support staff (including animal care technicians and licensed veterinary technicians) may increase the capacity at any given clinic, and so it is a better measure of the true supply. The vintage of the data used was January of 2020 for the clinic employee count and April of 2020 for the clinic location [41].

This research used the 2019 household count which was updated last by ESRI in June of 2019 [42]. In describing their methodology for accumulating and aggregating household count data, ESRI indicates that they use an approach based off United States Postal Service delivery route data which is then aggregated by the various geographic units of analysis [43]. This variable was used as the basis to evaluate the provider supply ratio for veterinary staff.

### 2.3. Human Health Care Service Supply

Human health care service here were conceptualized as the ratio of PCPs to the human population within the census tract. The data used for the primary care physicians was purchased from the Alabama Board of Medical Examiners & Medical Licensure Commission on February 24, 2021. Both MD and DO physicians were selected to be included in the data across the study area. The PCP acts as the entry point for a patient’s health and medical needs, and they can help navigate the healthcare system on the patient’s behalf [2]. PCPs can specialize in different fields of medicine. According to the American Academy of Family Physicians [2], a PCP specializes in Internal Medicine, Family Medicine, or Pediatrics. However, the OB-GYN can also act as the PCP as they have ongoing contact with their patients and provide counseling and medical advice [44]. While there are other specialties a PCP can choose to pursue, the specialties of Internal Medicine, Family Medicine, Pediatrics, and OB-GYN were available to be purchased by the Alabama Board of Medical Examiners & Medical Licensure Commission for the four counties and represent a foundational PCP workforce for this analysis.

Esri’s ArcGIS Online was used to obtain the 2020 Total Population data for the four Alabama counties. The 2020 Total Population data used point estimates representing July 1 of the current year [45].

### 2.4. Enhanced Two-Step Floating Catchment Area Model

The Enhanced Two-Step Floating Catchment Area model was used to evaluate the supply of both veterinary staff and PCPs in the study area.

For step 1, I determined ‘service catchments’: the provider-to-population ratio within a catchment area of each service provider at three different driving times (10 min, 20 min, and 30 min) using AGOL Online. In this case, I based the catchment sizes on empirical data reported by Neal and Greenberg [46]. Traffic impedance was not included in the drive time calculations due to time-of-day variances in order to obtain a more generic average drive time. Number of veterinary staff and household count were used for the veterinary access while the 2020 total population and MD and DO physicians who specialized in Internal Medicine, Family Medicine, OB-GYN, or Pediatrics were used for the PCP access. Populations were represented by census tract centroids. A distance decay was applied to each of the drive time bands based on a stepwise Gaussian function weighting scheme [25,47,48].

Step 1 is expressed as:

$$R_{j}=\frac{S_{j}}{\sum_{k\in \left\{d_{kj}\in D_{r}\right\}}P_{k}W_{r}}=\frac{S_{j}}{\sum_{k\in \left\{d_{kj}\in D_{1}\right\}}P_{k}W_{1}+\sum_{k\in \left\{d_{kj}\in D_{2}\right\}}P_{k}W_{2}+\sum_{k\in \left\{d_{kj}\in D_{3}\right\}}P_{k}W_{3}}$$

Step 2 determines the ‘population catchment’ or the ‘allocation’ of service ratios to the populations [49]. This was done by identifying and aggregating providers located within each population’s catchment area and applying the same distance decay.

Step 2 is expressed as:

$$A_{i}^{F}=\underset{j\in \left\{d_{ij}\in D_{r}\right\}}{\sum }R_{j}W_{r}=\underset{j\in \left\{d_{ij}\in D_{1}\right\}}{\sum }R_{j}W_{1}+\underset{j\in \left\{d_{ij}\in D_{2}\right\}}{\sum }R_{j}W_{2}+\underset{j\in \left\{d_{ij}\in D_{3}\right\}}{\sum }R_{j}W_{3}$$

where: *j* = catchment area; *k* = population/households census tract centroids within a travel time zone;$\;D_{r}\;$ = travel time zone from location *j*; $R_{j}\;$ = weighted physician-to-population ratio and weighted veterinary staff to household population; $P_{k}$ = the population/households of census tract *k* falling within the catchment; $S_{j}$ = the number of physicians/veterinary staff at location *j*; $d_{kj}\;$ = the travel time between *k* and *j*; $W_{r}$ = the distance weight for the *r*th travel time zone calculated from the Gaussian function (captures the distance decay of access to the PCP or the veterinary staff); $A_{i}^{F}\;$ = access to primary care physicians/veterinary staff for the population/households at location *i*; $d_{ij}$ is the travel time between *j* and *i.*

*Step 1 and 2 follow the methodology as advanced by Luo and Qi [25].

The results of the E2FSCA were then mapped as choropleths to indicate the related decayed supply-to-population ratio across each census tract in the four-county study area. Secondly, the data can be reviewed statistically by plotting the distance decayed sum supply-to-demand ratio on a scatterplot. A Pearsons r correlation coefficient was then calculated to quantify the degree to which the values are correlated.

## 3. Results

**Figure 2:**
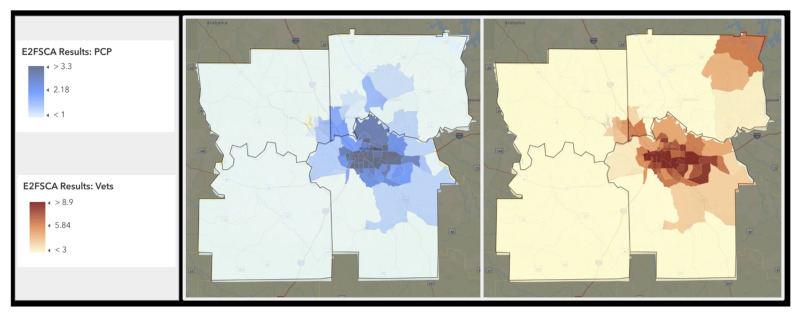
Results of the E2SFCA for PCPs (**left**) and veterinary staff (**right**). Similar spatial patterns in care access can be seen between both types of care providers. Note that darker colors denote relative higher levels of care access.

The choropleths summarizing the two resulting access to care variables are presented in Figure 2. The left portion of the figure displays the decayed supply of primary health care providers while the righthand section of the figure displays the decayed supply of veterinary care providers.

**Figure 3:**
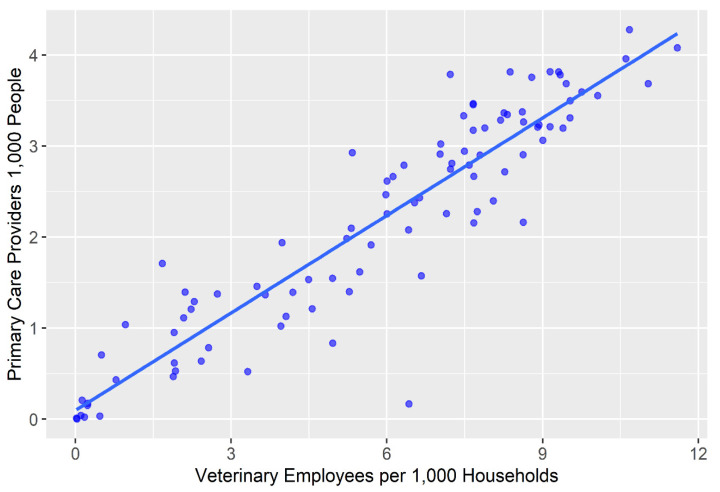
Scatterplot showing the relationship between the results of the E2SFCA for veterinary staff and PCPs.

A scatterplot is provided in Figure 3 which shows the relationship between the PCP results and the veterinary care results as well as a line of best fit.

A Pearsons r correlation coefficient was calculated to evaluate the relationship. The relationship between the supply of veterinary staff and the supply of primary care *physicians was found to be strongly positive r(93) = 0.92, p < 0.001 where n = 95.*

## 4. Discussion

Co-occurrence in lower access to care expressed both in terms of access to PCPs and access to veterinary staff is evaluated in two ways. The maps can be visually inspected to note the higher concentrations of access in the metropolitan areas with lower access in the rural regions. Census tract size is a relative indicator of population. Tracts that cover larger areas generally have lower population density, thus the large tracts surrounding the metropolitan Montgomery region indicate areas of more rural population characteristics. It is apparent from the maps that there is much similarity in the census tracts whether looking at the availability of veterinary care providers or primary care providers. Note higher values indicate relatively better access. There is an anomaly in the Northwest portion of the study area where higher numbers of veterinary care providers can be found. These results were validated against the point level clinic data. Indeed, there are several veterinary clinics located here though some are specialty facilities (acupuncture for example).

The scatterplot in Figure 3 as well as the Pearsons r results show the relationship between the results of the E2SFCA for veterinary care providers and the same output for the primary care providers by census tract in the four-county study area. A clear positive relationship is seen and confirms the impressions from the choropleth maps. Census tracts that have lower access to veterinary care similarly lack access to primary care physicians, indicating a potential concern when viewing care through a One Health paradigm.

The implications of this work are several. The first implication is the evidence that the E2SFCA can be used to examine access to care from a One Health perspective. The use of this approach for evaluating access to care across multiple disciplines expands the understanding of the true impact of access disparities. Including additional types of care providers (such as dentistry) could further add to the application of E2SFCA as a tool for identifying areas that lack access to care across a spectrum of care needs. Further combining other areas of supply and demand for veterinary care, such as the presence of animal agriculture to the regional supply of large animal veterinarians, would be another possible line of research. This work also highlights some of the specific limitations and potential advancements and refinements that could be applied by future researchers looking to use similar models to explore access to care.

A second implication is the identification of the areas in the four-county study area that are underserved by both PCPs and veterinary staff. This research clearly indicated that census tracts with low access to veterinary care also had low access to primary health care providers. With the potential for zoonotic diseases as well as the potential impact of lack of access to veterinary care on the psychosocial wellbeing of human caregivers, this finding is concerning. With the results of this study, policies could be designed to incentivize providers to locate in these areas of limited access to both veterinarians and primary care providers. Researchers and policy makers could make use of this combined E2SFCA methodology to evaluate other areas where an intersection of need in primary human and companion animal health co-occur and prioritize them for One Health interventions. Alternatively, integrated care centers which provide treatment to both humans and their animals may be an efficient way to address multiple disparities, including the use of combined mobile clinics in rural areas.

### Limitations and Future Research

The drive distance function used in the E2FSCA assumes equal decay across both rural and urban subzones. When using this model in smaller geographic areas, the specification of these catchment sizes is more critical than the specification of the distance decay function [50]. Neal and Greenberg found differences in average drive times to veterinary clinic by degree of rurality, where rural residents typically drive further distances to access veterinary care [46]. As discussed in the introduction, the assumption of equal distance decay across subzones is a known limitation of the E2FSCA. Some recent work in the banking sector using even more refined decay functions that account for human geography has shown some potential and could be explored in future research [51].

It has been found that the E2SFCA may tend to overestimate available resources with the possible introduction of a third step of spatial impedance to control for that overestimation [52]. Others have proposed that the stepwise function in the distance decay should be replaced with a continuous decay [53]. This is not seen as a significant limitation here since the goal was largely to show the relationship between access across humans and companion animals. As further research on veterinary care access is completed, it may be possible to expand this understanding to the Three-Step Floating Catchment Area model and its various recent improvements.

Using raster data would have provided improved accuracy. Updated US Census data in a raster format was not available at the time this research was conducted, with the only available raster data being over a decade old. Using census tract centroids has limitations since some locations had zero demand at 0–10 min distance band due to location of the tract centroid falling outside of that drive time polygon. Since the 10–20 min distance band is subjected to a distance decay, this artificially reduces the demand allocation. Similarly, this can over-represent supply in small tracts. Once updated raster data is available, it would be beneficial to use it in future analyses.

An additional limitation is inherent in the use of the number of households to represent the demand on veterinary service providers. Good models to estimate companion animal populations do not exist, particularly at the local level. This work assumes a constant number of animals per household and a constant proportion of pet owning households in order to compare access by tract. This assumption may be flawed, particularly due to the variance in human population density across the study area, one possible demographic factor that may influence pet ownership rates and number of pets in any given household [54]. It is worth noting, however, that the general assumption is that individuals in rural areas tend to have more animals and have a higher likelihood of having an animal in the household [54], which would only further highlight the patterns in disparity in access to veterinary care found in this research. Similarly, there may be some clinic staff attributed to care access in this study that are strictly large animal service providers or mobile providers. The overall number is likely not high enough to have a substantive impact on the analysis.

## 5. Conclusions

This research found strong correlation between the lack of access to human medical care and access to veterinary care. Given the acknowledgement in the One Health paradigm that human health and animal health are interconnected, these areas of dual deficiency are particularly concerning. This work highlights the need for policymakers to consider access to care across human and animal welfare in order to advance a One Health approach to wellness, particularly within a climate of heightened awareness of the potential for zoonotic disease impacts during the recent pandemic. It opens this area for future research to build upon. Lastly, this work advances the use of the E2SFCA as a model with potential for future research to examine other areas for concomitant lack of access in a One Health framework. Aims to improve various model parameters as well as the potential for deriving more complex aggregate scoring of access to various types of One Health care provide a wealth of opportunity for further application of this work.

## Data Availability

Census data are freely accessible online at data.census.gov. Locations of primary care providers can be purchased as described in the research and clinic data can be accessed through ESRI. Licensing restrictions prevent the distribution of both the PCP location data as well as the veterinary clinic location data.
